# Machine Learning Prediction of Intensive Care Unit Outcomes in Atrial Fibrillation Patients: A Rapid Review

**DOI:** 10.7759/cureus.99732

**Published:** 2025-12-20

**Authors:** Victoria Nguyen, Scot Garg, Rahul Mittal

**Affiliations:** 1 Department of Health Informatics, Rutgers University, New Brunswick, USA; 2 School of Medicine, University of Central Lancashire, Preston, GBR

**Keywords:** atrial fibrillation, intensive care unit, length of stay, machine learning, mortality prediction, predictive modeling

## Abstract

Atrial fibrillation (AF) in intensive care unit (ICU) patients is associated with higher mortality, longer stays, and greater resource use than in patients without AF. Machine learning may improve risk stratification in this high-risk population, but existing models have not been systematically synthesized. This rapid review summarizes how machine learning methods have been used to predict outcomes in ICU patients with AF, with primary emphasis on mortality and current gaps in length of stay (LOS) modeling.

Searches of PubMed, Embase, and Scopus (2015-2025) identified studies applying machine learning to intensive care outcomes in AF. Screening and data extraction were conducted in a web-based system using a single-reviewer approach with verification. Extracted items included study design, cohort characteristics, modeling approach, and performance metrics, and risk of bias and applicability were appraised using tools appropriate for prediction-modeling studies. Of 597 records screened, three studies met the inclusion criteria. All were US-based and used large electronic health record (EHR) datasets (sample sizes: 5,998-10,144). Algorithms evaluated included adaptive boosting, random forest, and stacking ensembles, with discrimination ranging from moderate to excellent for mortality prediction (area under the curve (AUC): 0.768-0.978). Frequently selected predictors included age, ICU severity indices (Acute Physiology Score III, Simplified Acute Physiology Score II, Sequential Organ Failure Assessment), vital signs, renal and metabolic laboratory values (e.g., blood urea nitrogen, estimated glomerular filtration rate, glucose), blood indices (such as white blood cell count and red cell distribution width), treatment indicators (mechanical ventilation, vasopressors, anticoagulation), and glycemic variability (GV). Steps toward clinical use were limited to prototype or web-based tool development, and routine deployment was not reported. Notably, none of the included studies developed or validated an LOS regression model.

Overall, machine learning shows clear promise for mortality prediction in ICU patients with AF, but implementation remains limited, and key operational outcomes remain understudied. Priorities for future work include external validation across diverse settings, prospective evaluation of clinical impact, development of models for additional resource and utilization outcomes alongside mortality prediction, and assessment of fairness across patient groups to support safe, equitable, and scalable clinical use.

## Introduction and background

Atrial fibrillation (AF) is one of the most common cardiac rhythm disturbances among intensive care unit (ICU) patients, occurring both in those with pre-existing AF and those who develop new-onset AF during critical illness [1,2]. ICU patients with AF have higher mortality, longer hospital stays, and more complications than those without AF [3,4]. The unstable physiological state of critically ill patients, together with hemodynamic instability, drug interactions, electrolyte imbalances, and the need for invasive monitoring, often reduces the effectiveness of traditional prediction tools [5-7]. New-onset AF develops in approximately 11% of critically ill medical ICU patients and is associated with prolonged ICU stays and higher mortality [8]. Postoperative AF occurs in about 20% of cardiac surgery patients and contributes to longer recovery times and increased healthcare resource use [2,9]. Given this burden, accurate risk stratification and outcome prediction for ICU patients with AF are clinically important.

Machine learning techniques have shown strong potential to address these challenges. Machine learning models can identify nonlinear relationships, account for time-dependent changes, and manage large, complex ICU datasets. Traditional ICU scores such as Sequential Organ Failure Assessment (SOFA) and Simplified Acute Physiology Score II (SAPS II) were not designed specifically for AF populations and may not fully capture rhythm-related factors, anticoagulation strategies, or temporally varying variables such as glycemic control, which has motivated interest in machine learning approaches [10-13]. At the same time, limited reporting of validation, calibration, and fairness raises questions about how ready existing models are for clinical use [7,14].

To address these gaps, we used a rapid-review approach, which follows the overall structure of a systematic review but streamlines steps such as protocol registration and dual screening to produce timely, decision-relevant evidence. This rapid review summarizes studies that developed or validated machine learning models predicting mortality and, where available, length of stay (LOS) in ICU patients with AF and compares model performance and key methodological features, with a focus on validation, calibration, interpretability, and fairness to assess their readiness for clinical application. In this review, we report discrimination using the area under the receiver operating characteristic curve (AUC), use “calibration” to refer to the agreement between predicted and observed risk, and note where studies used SHapley Additive exPlanations (SHAP) to support model interpretability. The research question is: among adult ICU patients with AF, how accurately do supervised machine-learning models predict short-term and longer-term mortality (e.g., in-hospital, 30-, 90-, and 360-day outcomes) and, where available, length of stay?

## Review

Methods

Eligibility Criteria (Population, Intervention, Comparison, and Outcome (​​​PICO))

Eligible studies enrolled adult patients (aged 18 years or older) admitted to intensive or critical care units with AF, including pre-existing AF, new-onset AF during the ICU stay, or postoperative AF requiring ICU admission (population). The studies had to develop or validate supervised machine learning models for predicting outcomes in ICU AF populations (intervention). When available, other prediction approaches within the same study, such as alternative machine learning models or traditional clinical risk scores, were considered as comparators, although the presence of a comparator was not mandatory for inclusion (comparator). The outcomes of interest were prognostic endpoints relevant to intensive care, with primary emphasis on mortality at different horizons (e.g., in-hospital, 30-, 90-, and 360-day mortality) and, where available, ICU or hospital LOS. We did not impose any geographic restrictions. However, several non-US studies identified in full-text screening were excluded because they either focused on postoperative AF outside the ICU, used risk-score approaches without supervised machine learning prediction, or targeted outcomes such as stroke, bleeding, or composite events rather than mortality or LOS in ICU AF cohorts.

Search Strategy

The search strategy was designed to capture all relevant studies applying supervised machine learning methods to predict outcomes among ICU patients with AF. A rapid, Preferred Reporting Items for Systematic Reviews and Meta-Analyses (PRISMA)-guided approach was used, and the review protocol was not registered. To ensure broad coverage and minimize the chance of missing relevant studies, three major databases, PubMed, Embase, and Scopus, were searched using tailored queries that combined terms for AF, ICU populations, and predictive modeling.

In PubMed, the query included the following: ("Atrial Fibrillation/epidemiology"[MeSH]) AND (("intensive care units"[MeSH Terms]) OR (intensive care units[Title/Abstract]) OR (icu[Title/Abstract])). In Embase, the search used included: ('atrial fibrillation'/exp OR 'atrial fibrillation') AND ('intensive care'/exp OR 'intensive care') AND ('predictive model'/exp OR 'predictive model' OR 'risk model'/exp OR 'risk model' OR 'machine learning'/exp OR 'machine learning'), limited to 2015-2025, articles, Embase/MEDLINE sources, and English. In Scopus, the query was as follows: TITLE-ABS-KEY (("atrial fibrillation" OR afib) AND ("intensive care" OR ICU) AND ("predictive model" OR "machine learning" OR "risk score")), filtered for 2015-2025, medicine, article type, and English. Searches were first run without date limits and then restricted to 2015-2025 to reflect contemporary applications of machine learning in critical care, aligning with the period when these methods became more common in healthcare research. All searches were completed in April 2025.

Study Selection

**Figure 1:**
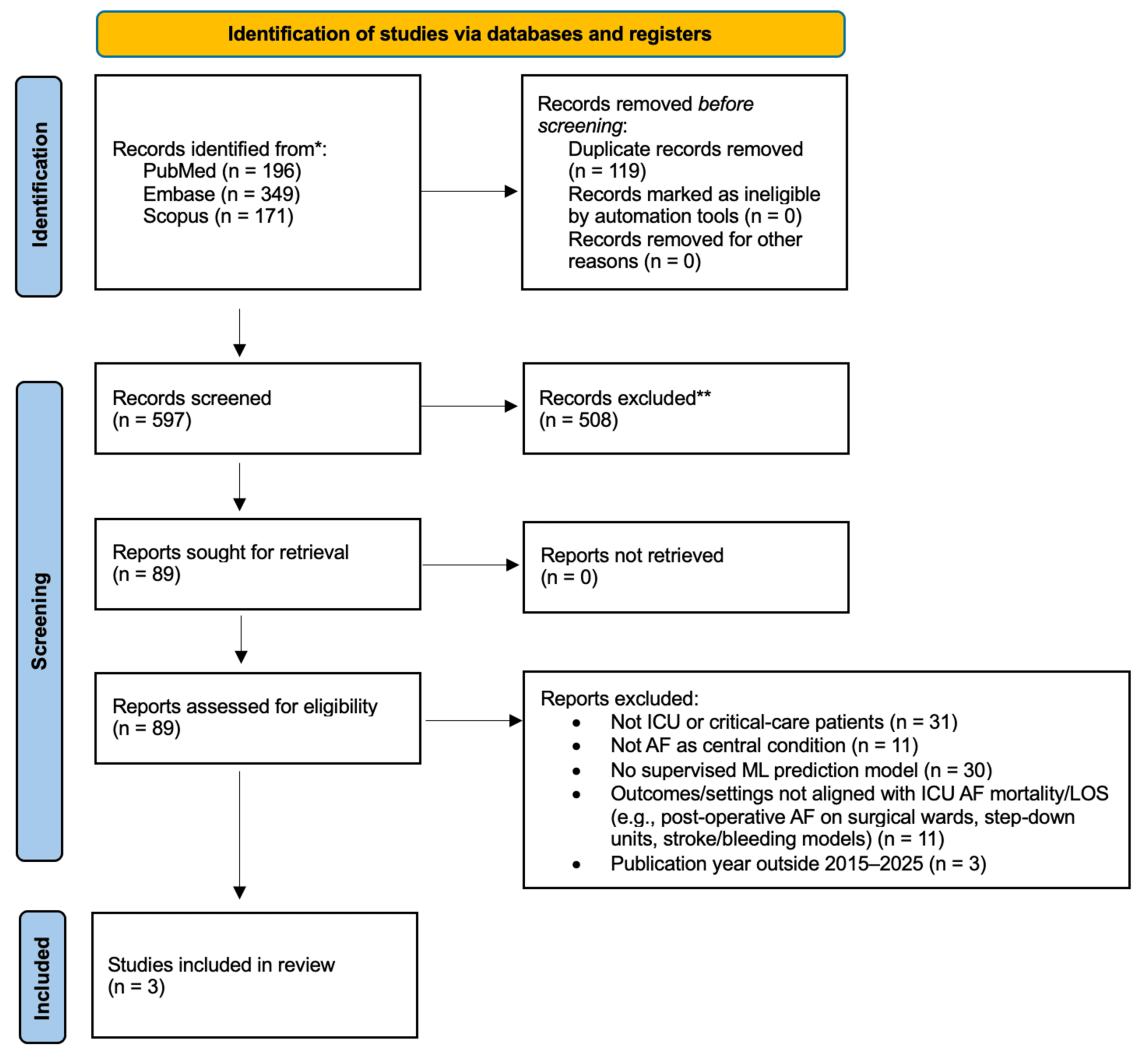
PRISMA flow diagram of study selection PRISMA: Preferred Reporting Items for Systematic Reviews and Meta-Analyses No geographic restrictions were applied; some non-US studies were excluded due to population, setting, or outcome mismatch rather than country

All records were screened in two stages (title/abstract, then full text) according to the predefined eligibility criteria described above. Full-text reasons for exclusion are summarized in Figure 1.

Data Extraction

We piloted a standardized extraction form. Study-level fields included author/year, design, setting/region, data source(s), period, cohort size, and patient descriptors (age, sex, AF status, comorbidities, admission context). Modeling fields included ICU outcomes (mortality and LOS), task type (classification; regression/time-to-event for LOS), algorithms, feature sets, validation strategy (internal split, cross-validation, external/temporal), and performance metrics on held-out data. We noted interpretability (e.g., SHAP [15]), clinical implementation/usability, and, when multiple models were tested, the best performer. We did not meta-analyze or re-benchmark across studies. We recorded whether models were used clinically, whether fairness was assessed [14], and whether external validation occurred. For LOS, we planned to record ICU length of stay when reported; in practice, none of the included studies developed or validated an LOS prediction model. For mortality, we captured the time horizon. All extractions were verified for consistency prior to synthesis.

Risk of Bias Assessment

We used the Prediction Model Risk of Bias Assessment Tool (PROBAST) to appraise included prediction-model studies [16]. PROBAST evaluates risk of bias and applicability across four domains: participants, predictors, outcome, and analysis. One reviewer completed the PROBAST assessment using a standardized form, and a second reviewer checked all judgments, with any disagreements resolved by consensus. Each study received an overall risk-of-bias judgment (low, high, or unclear) and an applicability rating (low, high, or unclear), with brief rationales. In addition, we assessed reporting completeness against the machine learning-specific reporting guidance and documentation standards outlined in PROBAST+AI [17], recording key items such as data source transparency, candidate predictor reporting, validation strategy, calibration details, code or model artifact availability, and subgroup performance reporting.

Results

Study Identification and Screening

We retrieved 716 records from the database searches: PubMed (n = 196), Embase (n = 349), and Scopus (n = 171). After removing 119 duplicates, 597 unique records remained for screening. Following abstract review, 508 records were excluded. A total of 89 full-text articles were assessed for eligibility, of which 86 were excluded for reasons such as not including ICU or critical-care patients (n = 31), not focusing on AF as a central condition (n = 11), absence of supervised machine learning prediction models (e.g., purely risk-factor analyses or traditional regression-based scores; n = 30), outcomes or settings not aligned with our predefined focus on mortality or LOS in ICU AF cohorts (e.g., postoperative AF on surgical wards, non-ICU or stepdown units, or prediction of stroke/bleeding without mortality modeling; n = 11). An additional three studies were excluded because the publication year fell outside the 2015-2025 window (n = 3). Ultimately, three studies met the inclusion criteria and were included in the final synthesis (Table 1). The study selection process followed PRISMA guidelines and is illustrated in Figure 1. All three studies were published in 2024. They represented different research teams and hospitals.

Included Study Overview (Designs, Settings, Algorithms)

All three development cohorts used MIMIC-IV; one study was also validated in eICU-CRD (multicenter). Algorithms spanned AdaBoost, LightGBM, and stacking. Table 1 summarizes each study’s setting, design, cohort sizes, model type, and data sources. Together, these studies represent general AF cohorts and a high-risk HF+AF subgroup, enabling comparison across clinical profiles and validation designs.

**Table 1:** Summary of included studies AF: atrial fibrillation; ICU: intensive care unit; LOS: length of stay; EHR: electronic health record; MIMIC-IV: Medical Information Mart for Intensive Care IV; eICU-CRD: eICU Collaborative Research Database

Year	Author	Disease context	Type of study	Number of patients	Intervention	Delivery method	Patient group
2024	Luo et al. [12]	Atrial fibrillation	Retrospective cohort	10,144 (development), 14,932 (validation)	AdaBoost machine learning model	Electronic health record data from MIMIC-IV and eICU-CRD	ICU patients with established AF
2024	Chen et al. [13]	Atrial fibrillation with focus on glycemic variability	Retrospective cohort	8,989 (primary), 837 (external validation)	LightGBM incorporating glycemic variability	Electronic health record data from MIMIC-IV	Critically ill ICU patients with AF
2024	Chen et al. [11]	Heart failure combined with atrial fibrillation	Machine learning prediction study	5,998 ICU patients	Stacking ensemble machine learning model (Random Forest, XGBoost, LightGBM, KNN)	Electronic health record data from MIMIC-IV	ICU patients with both heart failure and AF

Study Objectives

All included studies developed supervised machine learning models to predict mortality among ICU patients with AF. None developed or validated a LOS model, representing an actionable evidence gap for operational planning.

One study produced a compact, bedside-feasible 15-variable AdaBoost model [12]; another assessed glycemic variability (GV) as a prognostic feature [13]; and a third tested stacking ensembles in an HF + AF subgroup [11]. Two studies reported external/temporal validation; one reported internal validation only. Table 2 summarizes cohort size, age and sex distributions, and other key characteristics of each study.

**Table 2:** Participant characteristics AF: atrial fibrillation; ICU: intensive care unit; IQR: interquartile range; GV: glycemic variability; HF: heart failure; MIMIC-IV: Medical Information Mart for Intensive Care IV; eICU-CRD: eICU Collaborative Research Database; NR: not reported; LASSO: least absolute shrinkage and selection operator

Year	Title	Age range	Median/mean age	Sex (M/F)	Other relevant info
2024	A machine learning-based predictive model for the in-hospital mortality of critically ill patients with atrial fibrillation [12]	≥18 years	75.4 years (IQR 67.9-84.3)	M: 59.0%, F: 41.0%	MIMIC-IV development; in-hospital mortality 18.2%; external validation in eICU-CRD; web-based tool evaluated by 81 medical experts with >95% positive ratings
2024	Prognostic value of glycemic variability for mortality in critically ill atrial fibrillation patients and mortality prediction model using machine learning [13]	≥18 years	76.5 years (IQR 67.7-84.3)	M: 57.8%, F: 42.2%	Primary cohort: 8,989 AF ICU patients (2008-2019); temporal external cohort: 837 (2020-2022); 30-day mortality 23.5%, 90-day 31.0%, 360-day 41.0%; GV calculated from blood glucose (median sampling interval ≈11.7 hours)
2024	Predicting in-hospital mortality in patients with heart failure combined with atrial fibrillation using stacking ensemble model: an analysis of the medical information mart for intensive care IV (MIMIC-IV) [11]	≥18 years	NR	NR	High-risk HF+AF ICU population (n=5,998); in-hospital mortality 14.86%; MIMIC-IV 2008-2019; 22 features selected via Mann-Whitney U and LASSO; no external validation

Participant Cohorts and Sample Sizes

Although our eligibility criteria encompassed pre-existing AF, new-onset AF during ICU admission, and postoperative AF requiring ICU admission, the included studies predominantly analyzed ICU admissions with documented AF at or before ICU admission. Across all included studies, AF was identified using structured diagnostic codes in the underlying ICU databases (ICD-9 427.31 and ICD-10 I48.x). None of the studies distinguished pre-existing versus new-onset AF through rhythm-monitoring adjudication, and AF timing was not separately validated. Luo et al. [12] and Chen et al. [13] focused on critically ill patients with AF recorded in MIMIC-IV, while Chen et al. [11] restricted their cohort to a higher-risk subgroup with both heart failure and AF. None of the three studies specifically targeted isolated postoperative AF populations. As a result, the synthesized models primarily reflect general AF and HF+AF ICU cohorts, rather than distinct new-onset or strictly postoperative AF groups.

Data Sources and Feature Domains

All studies used de-identified electronic health record (EHR) ICU databases: Medical Information Mart for Intensive Care IV (MIMIC-IV) for development, the eICU Collaborative Research Database (eICU-CRD) for cross-hospital validation (one study), and a temporal external cohort within MIMIC-IV (one study). Extracted domains included demographics, ICU context, vital signs (heart rate (HR), blood pressure (BP), respiratory rate (RR), oxygen saturation (SpO₂)), laboratory values (renal/metabolic panels, blood counts, glucose), severity indices (Acute Physiology Score III (APS III), Simplified Acute Physiology Score II (SAPS II), and Sequential Organ Failure Assessment (SOFA)), comorbidities, and interventions (mechanical ventilation (MV), renal replacement therapy (RRT), anticoagulation). One study engineered GV from serial glucose values [13].

Modeling Approaches

Luo et al. [12] compared 10 algorithms and selected AdaBoost, optimized with Bayesian search over 52 iterations, and developed both a full model and a compact 15-variable model, validated internally in MIMIC-IV and externally in eICU-CRD. Chen et al. [11] implemented a stacking framework combining RF, XGBoost, LightGBM, and KNN with CatBoost as the meta-learner, using Mann-Whitney U followed by LASSO to select 22 features, and compared the stack against single models and SOFA/SAPS II. Chen et al. [13] evaluated seven algorithms, identified LightGBM as the best performing, and showed that GV ranked among the top predictors, with temporal external validation and deployment as a web tool. Across studies, boosted trees and ensemble methods predominated, consistent with contemporary machine learning practice [12,13].

Training and validation procedures were only partially reported. Luo et al. [12] randomly split MIMIC-IV into development and internal-validation cohorts and used Bayesian or grid-based search to tune hyperparameters before external validation in eICU-CRD; however, the handling of class imbalance and missing data was described briefly and without formal sensitivity analyses. Chen et al. [13] trained LightGBM models on a 2008-2019 AF cohort and performed temporal validation in a 2020-2022 cohort, but did not provide detailed information on resampling strategies or class-imbalance methods beyond standard LightGBM settings. Chen et al. [11] used a training/test split within MIMIC-IV for stacking and single-model comparisons; missing-data handling and hyperparameter tuning were described at a high level; and no resampling techniques for imbalance were explicitly reported. None of the three studies used nested cross-validation or bootstrap resampling for optimism correction.

Predictive Performance, Algorithms, and Key Predictors

Across the three included studies, machine learning models demonstrated moderate to excellent discrimination for mortality (AUC: 0.768-0.978). Mortality horizons differed: Luo et al. [12] predicted in-hospital mortality, Chen et al. [11] also predicted in-hospital mortality in a HF + AF subgroup, and Chen et al. [13] predicted 30-, 90-, and 360-day mortality. Boosted-tree and ensemble approaches (AdaBoost, LightGBM, stacking) were the dominant algorithms, and all relied on routinely available ICU variables, including age, vital signs, renal and metabolic markers, and severity scores (APS III, SOFA, SAPS II). Study-specific predictors included anticoagulation regimen [12] and GV [13], the latter emerging as a strong, clinically actionable feature. Confidence intervals were inconsistently reported, and none of the studies provided formal calibration metrics. External or temporal validation improved generalizability in two studies, whereas the stacking model lacked external testing. Overall, the models performed well within individual cohorts but varied in completeness of reporting and calibration transparency. Table 3 summarizes the outcome horizons, algorithms, key predictors, discrimination (with 95% confidence intervals where reported), and calibration reporting for each included study.

**Table 3:** Study outcomes NR: not reported; BUN: blood urea nitrogen; AF: atrial fibrillation; HF: heart failure; ICU: intensive care unit; AUC: area under the curve; HR: heart rate; BP: blood pressure; RR: respiratory rate; SpO₂: peripheral oxygen saturation; APS III: Acute Physiology Score III; SOFA: Sequential Organ Failure Assessment; SAPS II: Simplified Acute Physiology Score II; CHA₂DS₂-VASc: congestive heart failure, hypertension, age ≥75 years (doubled), diabetes, prior stroke/TIA (doubled), vascular disease, age 65-74 years, sex category; MELD-Na: model for End-Stage Liver Disease–sodium, WBC: white blood cell count, BUN: blood urea nitrogen, BMI: body mass index, MV: mechanical ventilation; RRT: renal replacement therapy; GV: glycemic variability; ACEI/ARB: angiotensin-converting enzyme inhibitor/angiotensin receptor blocker; RF: random forest; XGB: Extreme Gradient Boosting; KNN: k-nearest neighbors None of the included studies reported quantitative calibration metrics (e.g., calibration slope, intercept, or Brier score)

Year	Study	Outcome horizon	Algorithm(s)	Key predictors (abbrev.)	Internal AUC (95% CI)	External/temporal AUC	Calibration reporting	Notes
2024	Luo et al. [12]	In-hospital mortality	AdaBoost (best of 10)	Age; HR/BP/RR/SpO₂; APS III; CHA₂DS₂-VASc; MELD-Na; WBC; BUN; creatinine; electrolytes; comorbidities; anticoagulation regimen	0.978 (0.970–0.986) full; 0.977 (NR) compact	0.825 (0.801-0.849) full; 0.807 (NR) compact	No slope/intercept/Brier; no calibration plot	Compact 15-variable model performed similarly to full; web-based tool rated >95% favorable by clinicians
2024	Chen et al. [13]	30-, 90-, 360-day mortality	LightGBM	Glycemic variability (coefficient of variation); age; vital signs; SOFA; SAPS II; labs; comorbidities; MV; RRT; ACEI/ARB; beta-blocker; anticoagulants	0.780 (NR)	0.788 (NR) temporal	No quantitative calibration metrics; no plots	GV was a top predictor; ~20% GV threshold suggested; web tool deployed
2024	Chen et al. [11]	In-hospital mortality (HF+AF subgroup)	Stacking (RF + XGB + LightGBM + KNN; CatBoost meta-learner)	22 features: age; BMI; HR; BP; RR; SpO₂; SOFA; SAPS II; labs; comorbidities; treatments	0.768 (0.740-0.796)	-	Calibration plot only; no metrics	Outperformed single models and SOFA/SAPS II; no external validation

Clinical Implementation and Real-World Application

Evidence of clinical implementation was limited, with most work remaining at the development and validation stage. Two studies moved closer to bedside use by releasing web-based tools: Luo et al. [12] created an interface for their compact AdaBoost model and reported that 81 clinicians rated it favorably in more than 95% of evaluations for accuracy, interpretability, credibility, and usability, while Chen et al. [13] provided an online LightGBM-based platform that generates mortality risk estimates and displays feature contributions using SHAP visualizations [15]. In contrast, Chen et al. [11] did not describe any clinical deployment of their stacking ensemble. None of the models were reported as fully integrated into EHR systems with ongoing monitoring. Moving from promising performance metrics to routine clinical use will require EHR integration, staff training, governance for monitoring and recalibration, management of alert burden, and clear plans for long-term maintenance [18]. Overall, current evidence suggests that although machine learning models for ICU patients with AF show strong predictive performance, further work is needed to demonstrate feasibility, scalability, and sustained clinical impact in real-world practice.

Fairness and Equity Considerations

All three studies reported cohort demographics but varied in how demographic variables were used as predictors. Luo et al. [12] and Chen et al. [11] included sex and race or ethnicity in their feature sets, whereas Chen et al. [13] limited demographic inputs to age (and BMI) and did not model sex or race or ethnicity explicitly. Two cohorts were predominantly White, with approximately 74% of participants identified as White in Luo et al. [12] and Chen et al. [13], while the race distribution in Chen et al. [11] was less clearly described. None of the studies reported subgroup performance, such as AUC or calibration stratified by sex, race or ethnicity, or age. As a result, equity and potential differential performance across patient groups remain largely unassessed [14], highlighting an important priority for future research. With appropriate validation, calibration, and fairness evaluation, machine learning models may in the future contribute to more consistent and equitable risk stratification in ICU AF care, but current evidence is insufficient to confirm equitable performance across patient groups.

Risk of Bias and Reporting Quality (PROBAST and PROBAST+AI)

Using PROBAST, two studies (Luo et al. [12] and Chen et al. [13]) were judged to have low risk of bias in the participants, predictors, and outcome domains but were rated as having some concerns in the analysis domain, primarily due to sparse calibration reporting and lack of fairness assessment. The stacking study by Chen et al. [11] was rated as high risk of bias in the analysis domain (and overall), reflecting the absence of external validation and the limited quantitative calibration reporting. PROBAST+AI highlighted additional transparency gaps across all three studies, including the absence of calibration slope, intercept, and Brier score, unreported subgroup performance, and incomplete availability of code or model artifacts for reproducibility. Table 4 summarizes the domain-level PROBAST and PROBAST+AI assessments.

**Table 4:** Quality assessment checklist (prediction-model focus) ROB: risk of bias; NR: not reported; AF: atrial fibrillation; HF: heart failure; ICU: intensive care unit; APS III: Acute Physiology Score III; SOFA: Sequential Organ Failure Assessment; SAPS II: Simplified Acute Physiology Score II “Applicability (concern)” reflects concern regarding applicability to the review question. All studies lacked quantitative calibration metrics (e.g., calibration slope, intercept, or Brier score) and subgroup performance reporting per PROBAST+AI criteria

Study	Participants ROB	Predictors ROB	Outcome ROB	Analysis ROB	Overall ROB	Applicability (concern)	Key PROBAST+AI reporting gaps
Luo et al. (2024) [12]	Low	Low	Low	Some concerns	Low-some concerns	Low concern (US ICU AF)	No calibration slope/intercept/Brier; no subgroup performance; no model artifact availability
Chen et al. (2024) [13]	Low	Low	Low	Some concerns	Low-some concerns	Low concern (US ICU AF)	No calibration metrics; subgroup performance not reported; limited transparency on model internals
Chen et al. (2024) [11]	Low	Low	Low	High	High	Low concern (US HF+AF ICU subgroup)	No quantitative calibration metrics; no external validation; no subgroup performance; limited code/model reproducibility

Discussion

This rapid review synthesizes machine learning applications for AF in ICUs across three recent US studies (all 2024) and identifies a consistent pattern: boosted trees and ensemble algorithms achieve clinically useful discrimination for mortality, yet translation to routine care remains limited [11-13]. Crucially, no included study developed or validated an LOS regression model, leaving an important operational outcome unmet.

Summary of Main Findings

Ensemble and boosting methods captured meaningful risk signals in ICU AF. Luo et al. [12] identified AdaBoost as best among ten algorithms, with excellent internal AUCs and solid external validation; their compact 15-variable model maintained near-equivalent accuracy. Chen et al. [11] showed that stacking outperformed single models and traditional scores (SOFA/SAPS II), though without external validation. Chen et al. [13] demonstrated LightGBM stability across temporal cohorts and highlighted glycemic variability (GV) as a top predictor with an actionable ~20% threshold.

Across the three studies, discrimination (AUC: 0.768-0.978) ranged from moderate to excellent. Within individual cohorts, the machine learning models generally outperformed traditional scores such as SOFA and SAPS II, although the small, heterogeneous evidence base does not allow formal ranking of algorithms across settings. External or temporal validation reduced optimism while maintaining clinical relevance, underscoring the value of independent testing before deployment. Parsimonious models using routine variables enhance feasibility and resilience to missing data. Collectively, these findings support machine learning as a decision-support adjunct, not a replacement for clinical judgment.

Implementation Challenges and Clinical Translation Barriers

Despite encouraging performance and prototype web tools, none of the studies reported sustained, in-EHR deployment [11-13]. Barriers include EHR integration, clinician workflow fit, alert burden, and governance for recalibration and monitoring. These challenges can be addressed through structured implementation science frameworks but require investment, multidisciplinary collaboration, and institutional readiness [16-20].

Methodological Problems and Evaluation of Quality

Two studies validated their models on independent datasets, including geographic or temporal cohorts, strengthening generalizability relative to single-split designs. However, several gaps limit bedside interpretability: incomplete reporting of cohort construction (e.g., repeat ICU admissions), inconsistent predictor timing windows, limited calibration detail, and absence of decision-impact analyses such as decision curves or workload effects [19,20]. The limited reporting of calibration metrics and decision-curve analyses represents a key barrier to assessing whether these models provide well-calibrated risk estimates and net clinical benefit at relevant decision thresholds. Addressing these limitations will enhance credibility, transparency, and clinical adoption.

The methodological gaps described in this review, such as limited calibration reporting, absence of decision-impact analyses, and unassessed fairness, primarily relate to PROBAST+AI transparency and reporting items rather than classical internal-validity domains [16,17]. Accordingly, we rated two studies as having low risk of bias across most PROBAST domains but “some concerns” in the analysis domain, and one study [11] as high risk due to lack of external validation and sparse calibration detail.

Algorithmic Fairness and Bias Considerations

None of the studies reported subgroup performance (discrimination or calibration) by race/ethnicity, sex, or age. Equity and representativeness, therefore, remain unassessed, underscoring the need for pre-specified subgroup audits, per-group performance metrics, and postdeployment monitoring for differential performance [14]. When disparities arise, practical steps include threshold adjustment, subgroup-specific recalibration, or targeted model updates, accompanied by transparent documentation and public reporting. Such practices align with current expectations for trustworthy and fair AI in healthcare [14,18].

Comparison with Existing Literature

The findings of this review are consistent with other published work showing that machine learning is increasingly being used to predict outcomes in critically ill patients with AF and related ICU populations. Karri et al. [2] studied 6,349 ICU admissions after cardiac surgery and used several machine learning models to predict postoperative AF; their best model, a gradient boosting machine, achieved an AUC of 0.74 using routine preoperative data and outperformed the POAF score (AUC: 0.63). Guan et al. [3] developed an interpretable model for new-onset AF in critically ill patients using MIMIC-IV for training and a MIMIC-III cohort for external validation; their XGBoost model achieved an AUC of 0.891 in internal validation and 0.769 in external validation. Hong et al. [10] reported a scoring model for ICU stay and mortality in emergency AF admissions using a large EHR cohort, further illustrating the use of high-dimensional ICU data for risk prediction. In the three mortality-focused studies summarized in this review, stacking and LightGBM models similarly achieved higher AUCs than SOFA and SAPS II in AF-related ICU populations [11-13].

Two common themes appear across this literature. First, parsimony: models that use a relatively small number of routinely collected variables, such as the 15-variable AdaBoost model developed for critically ill AF patients [12], can maintain strong performance while being easier to implement and more robust to missing data [10]. Second, interpretability: methods such as SHAP values [15], clear clinical cut-points (e.g., GV around 20% [13]), and compact feature sets make model outputs easier for clinicians to understand and trust, which supports real-world usability and aligns with calls for interpretable approaches in high-stakes settings [18].

Clinical Implications and Future Directions

Machine learning models for ICU AF have the potential to support mortality risk stratification, but current evidence is insufficient for clinical deployment. Before implementation, models should undergo local “silent” validation to assess calibration, stability, and workflow fit. Institutions can then integrate calibrated predictions into dashboards or hand-off tools, accompanied by routine performance monitoring. Future research should incorporate decision-curve analysis to determine whether model-guided decisions provide net clinical benefit compared with existing practice [19,20].

To ensure fairness and transparency, future AF ICU studies should pre-specify key demographic subgroups, report subgroup-specific discrimination and calibration, and provide at least one global calibration metric (e.g., Brier score, calibration slope, and intercept). When substantial performance differences arise, mitigation strategies, such as subgroup-specific recalibration, should be reported. Broader external validation in diverse ICU settings and prospective evaluations will be essential to determine clinical utility and operational impact.

Strengths, Limitations, and Future Research

This rapid review followed a structured, multidatabase search strategy and applied PROBAST and PROBAST+AI to evaluate reporting quality and risk of bias [16,17]. The included studies consistently used routinely available ICU predictors and demonstrated the feasibility of boosted-tree and ensemble models in AF-related critical illness.

Several limitations must be noted. Only three eligible studies were identified, all retrospective and US-based, limiting generalizability to other health systems. Because all three cohorts were drawn from large US tertiary ICUs, the findings may not generalize to smaller or community ICUs with different case-mix, resource availability, and practice patterns. Although no geographic restrictions were applied, several non-US full-text articles were excluded because they focused on postoperative AF outside the ICU, general AF populations, or nonmortality outcomes such as stroke or bleeding. Calibration metrics and subgroup performance were rarely reported, and no study developed an LOS model. As a rapid review, some methodological steps were streamlined, and we did not search machine learning conference proceedings where relevant work may be published first.

Future work should emphasize multicenter external validation, reporting of calibration and fairness metrics, and assessment of clinical and operational impact. Prospective studies incorporating health-economic outcomes and real-world workflow evaluation are needed to support safe and scalable implementation.

## Conclusions

Machine learning models for ICU AF show promising discrimination for mortality prediction, with clinically plausible and, in some cases, novel predictors. However, the current evidence base is small, retrospective, and limited to a few US datasets, with sparse reporting of calibration, fairness, and real-world deployment. At this stage, machine learning models should be viewed as promising tools to support risk stratification and potentially inform clinical decision-making, rather than as proven interventions to improve patient outcomes. Prospective, multicenter evaluations are needed to determine their clinical utility, impact on decision-making, and effects on patient outcomes.
